# SOD2 immunoexpression predicts lymph node metastasis in penile cancer

**DOI:** 10.1186/s12907-015-0003-7

**Published:** 2015-03-03

**Authors:** Lara Termini, José H Fregnani, Enrique Boccardo, Walter H da Costa, Adhemar Longatto-Filho, Maria A Andreoli, Maria C Costa, Ademar Lopes, Isabela W da Cunha, Fernando A Soares, Luisa L Villa, Gustavo C Guimarães

**Affiliations:** Santa Casa de São Paulo, INCT-HPV at Santa Casa Research Institute, School of Medicine, Rua Marquês de Itú, 381, 01223-001 São Paulo, Brazil; Teaching and Research Institute, Barretos Cancer Hospital, Rua Antenor Duarte Vilela, 1331, 14784-006 Barretos, Brazil; Department of Microbiology, Institute of Biomedical Sciences, University of São Paulo, Av. Prof. Lineu Prestes, 1374 - Ed. Biomédicas II, Cidade Universitária, 05508-900 São Paulo, Brazil; Pelvic Surgery Department, A. C. Camargo Cancer Center, Rua Prof. Antônio Prudente 211, 01509-010 São Paulo, Brazil; Laboratory of Medical Investigation (LIM) 14, Department of Pathology, School of Medicine, University of São Paulo, Av. Dr. Arnaldo 455, 01246-903 São Paulo, Brazil; Life and Health Sciences Research Institute, School of Health Sciences, ICVS/3B’s - PT Government Associate Laboratory, University of Minho, Braga, Guimarães, Portugal; Molecular Oncology Research Center, Barretos Cancer Hospital, Pio XII Foundation, Barretos, Rua Antenor Duarte Villela, 1331, 14784-400 Barretos, Brazil; Department of Anatomic Pathology, A. C. Camargo Cancer Center, Rua Prof. Antônio Prudente 109, 01509-900 São Paulo, Brazil; Department of Radiology and Oncology, School of Medicine, University of São Paulo and Cancer Institute of the State of São Paulo, ICESP, Av Dr Arnaldo 250, 01246-000 São Paulo, Brazil

**Keywords:** Penile cancer, Superoxide Dismutase-2, Lymph node metastasis

## Abstract

**Background:**

Superoxide dismutase-2 (SOD2) is considered one of the most important antioxidant enzymes that regulate cellular redox state in normal and tumorigenic cells. Overexpression of this enzyme in lung, gastric, colorectal, breast cancer and cervical cancer malignant tumors has been observed. Its relationship with inguinal lymph node metastasis in penile cancer is unknown.

**Methods:**

SOD2 protein expression levels were determined by immunohistochemistry in 125 usual type squamous cell carcinomas of the penis from a Brazilian cancer center. The casuistic has been characterized by means of descriptive statistics. An exploratory logistic regression has been proposed to evaluate the independent predictive factors of lymph node metastasis.

**Results:**

SOD2 expression in more than 50% of cells was observed in 44.8% of primary penile carcinomas of the usual type. This expression pattern was associated with lymph node metastasis both in the uni and multivariate analysis.

**Conclusions:**

Our results indicate that SOD2 expression predicts regional lymph node metastasis. The potential clinical implication of this observation warrants further studies.

**Electronic supplementary material:**

The online version of this article (doi:10.1186/s12907-015-0003-7) contains supplementary material, which is available to authorized users.

## Background

Malignant penile tumors are rare in developed countries but exhibit relatively high prevalence in some developing countries. Regional lymph node metastasis is one of the most important prognostic factors in patients with penile carcinoma due to its correlation with the advanced pathological stage of the tumor and tumor-related death [1]. Other factors affecting prognosis are histological grade, tumor thickness, perineural invasion, lymphovascular invasion and pattern of invasion [2,3].

About 50% of the cases in which palpable suspicious lymph node were present, subsequent pathologic analysis failed to find any evidence of metastatic disease in the lymph nodes [4,5]. Conversely, 20% of lymph nodes with no clinical signal of disease display micro metastases [6]. Therefore there is a need to identify other markers that may predict the occurrence of inguinal metastasis, perineural and vascular invasion. The use of these markers could be valuable to better define the subset of patients that will benefit from different therapeutic approaches [2-7].

Several studies have shown that superoxide dismutase 2 (SOD2 or manganese superoxide dismutase) protein expression is up-regulated in colorectal, lung, gastric/esophageal, and cervical cancer cells when compared to normal tissues [8-11]. However, the relationship between SOD2 expression and penile cancer has not been addressed, mainly in terms of regional lymph node metastasis. This study aimed to evaluate the association of SOD2 immunoexpression with inguinal lymph node metastasis and its clinical implication.

## Methods

### Tissue samples

Penile samples from 125 patients were obtained from the Department of Anatomic Pathology, Medical and Research Center, A. C. Camargo Cancer Center, São Paulo, Brazil. No patient had distant metastasis at the diagnosis and all of them underwent tumor resection between 1953 and 2000. Lymphadenectomy has been performed in 50.4% of the cases and no patient received postoperative radiotherapy. Pathologic T stage was classified according to the TNM system of the International Union Against Cancer, 7th edition [12]. Ethical approval for this study was granted by the Hospital A.C. Camargo Institutional Research Ethics Committee (Project Number 1369/10).

Lymph node status has been defined using the pathologic information from the lymphadenectomy performed in 63 men (pN status). Patients who had not undergone lymph node resection had their lymph node status based on a retrospective longitudinal analysis of regional recurrence. Since no patient received inguinal or pelvic radiation as part of the treatment, those cases with no lymphadenectomy and no regional recurrence in a 3-year follow-up period had their lymph node status classified as “negative” (n = 46). Nevertheless, nine men without lymphadenectomy developed lymphonodal metastatic recurrence during follow-up after penectomy (median time to recurrence: 7.1 months; range: 1.4 – 22.1 months) and consequently they were considered as having “positive nodes” (tumor progression not previously detected). It was not possible to clearly define the regional lymph node status in seven cases with no previous lymphadenectomy. Although they did not have regional recurrence, the follow-up period was less than three years (median follow-up: 24.1 months; range: 8.7 – 35.8 months). These samples were not included in the uni and multivariate analyses.

### Immunohistochemical SOD2 detection

After deparaffinization in xylene and rehydration, antigen retrieval was performed by incubation in boiling citrate buffer pH 6.0 for 20 minutes. Endogenous peroxidase activity was inactivated with 3% hydrogen peroxide. Nonspecific avidin-binding was also blocked (DAKO, X0590, Carpinteria, CA, USA). Samples were incubated with an anti-SOD2 polyclonal antibody (Santa Cruz Biotechnology, sc-18504, Santa Cruz, CA, USA), 1:100 in 1% bovine serum albumin-phosphate buffered solution for 30 minutes at 37°C and for 18 hours at 4°C. Slides were then incubated for 30 minutes at 37°C with biotinylated rabbit anti-goat IgG, (Vector, BA5000, Burlingame, CA, USA) diluted 1:500, followed by incubation for 30 minutes at 37°C with the complex streptavidin and peroxidase (StreptABComplex/HRP Duet Mouse/Rabbit, DakoCytomation, K0492, Glostrup, Denmark), diluted 1:200 and developed using 100 mg of 3,3’-diaminobenzidine tetrahydrochloride (Sigma, D-5637, St Louis, MO, USA), 6% H_2_O_2_ in dimethyl sulphoxide and counterstained with Harris' hematoxylin. Sections derived from an ovarian papillary serous adenocarcinoma were used as a positive control for SOD2 expression.

In order to verify the specimen quality for immunohistochemistry, all samples were also checked using a cytokeratin panel, which was positive in all cases. In addition, positivity rate of SOD2 expression was analyzed according to the year of patient’s admission (1950–1959; 1960–1969; 1970–1979; 1980–1989; 1990–2000) and no statistically significant difference was found. Thus, specimen quality was considered adequate for analysis.

### Evaluation of tissue staining

Positive immunohistochemical reactions were evaluated considering the percentage of stained tumoral cells. Only tissue cores with more than 25% of tumor cells were analyzed. The evaluation was performed by examining the tumor core under X200 magnification. For the statistical analysis, reactions were scored as exhibiting less than 50% stained cells or more than 50% stained cells, as previously reported by our group [11]. Immunohistochemical evaluation was performed independently and blindly by two observers (ALF and FAS). The very few discordant results were discussed by both observers and a final score was established.

### Statistical analysis

The casuistic has been characterized by means of descriptive statistics. Fisher’s exact test was employed to compare categorical variables in the univariate analysis. Exploratory logistic regression has been proposed to evaluate the independent predictive factors of inguinal lymph node metastasis. Variables with p value less than 0.10 were included in the multivariate model, which was conducted using a stepwise forward technique. The significance level was set at 5% for all tests.

### DNA extraction

For DNA extraction, several 5 μm sections of the paraffin-embedded samples were collected in 1.5-ml microtubes. Samples were treated with xylene and digested with proteinase-K according to standard protocols described previously [13]. The microtome blade was changed after each block was cut and all the surrounding area and apparatus were cleaned with xylene and ethanol after processing each sample to avoid contamination between the samples. DNA quality was checked by amplification of the human β-globin gene using PCO3+/PCO4+ primers [14].

### HPV typing

HPV detection and genotyping using generic primers (GP5+/GP6+) and type specific probes (6, 11, 16, 18, 31, 33, 35, 39, 42, 45, 51, 52, 53, 54, 55, 56 and 58), respectively, was performed as previously described [15].

## Results

In the present study we have analyzed by immunohistochemistry the association of SOD2 expression pattern with different tumor aggressiveness and progression variables in 125 primary SCC samples of the usual histological type. Characterization of the population studied is depicted in Table 1. A representative immunostaining for SOD2 in different penile tumor samples exhibiting <50% or >50% stained cells is presented in Additional file 1. Most of the cases was classified as pT2 or pT3 (87.2%) and 44 cases had regional lymph node metastasis at diagnosis. No cases with distant metastasis were observed. Fifty six tumors (44.8%) had SOD2 expression greater than 50%. In about 20% of the cases HPV infection was detected [see Additional file 2]. No relationship was observed between presence of HPV DNA and SOD2 expression (Table 2).

**Table 1:** **Characterization of the population study (n = 125)**

**Variable**	**Category**	**N**	**%**
Age	<50 yo	43	34.4
	50 – 59 yo	41	32.8
	≥60 yo	41	32.8
pT (TNM)	pT1a	7	5.6
	pT1b	4	3.2
	pT2	52	41.6
	pT3	57	45.6
	pT4	5	4.0
Palpable suspicious regional lymph node	No	64	51.2
	Yes	59	47.2
	Unknown	2	1.6
Regional lymph node status	No metastasis	74	59.2
	Metastasis	44	35.2
	Unknown	7	5.6
Pattern of invasion	Pushing	14	11.2
	Infiltrating	101	80.8
	Unknown	10	8.0
Histological grade	Grade 1	15	12.0
	Grade 2	45	36.0
	Grade 3	65	52.0
Perineural invasion	No	83	66.4
	Yes	35	28.0
	Unknown	7	5.6
Vascular invasion	No	85	68.0
	Yes	33	26.4
	Unknown	7	5.6
Invasion of *corpora cavernous*	No	22	17.6
	Yes	60	48.0
	Missing data	43	34.4
Invasion of *corpora spongiosum*	No	4	3.2
	Yes	88	70.4
	Unknown	33	26.4
Invasion of urethra	No	42	33.6
	Yes	28	22.4
	Unknown	55	44.0
Tumor depth	≤5 mm	21	16.8
	>5 mm	92	73.6
	Unknown	12	9.6
HPV detection	No	99	79.2
	Yes	26	20.8
SOD2 expression	<50%	69	55.2
	>50%	56	44.8

**Table 2:** **Number and percentage of cases according to HPV genotype and SOD2 expression**

		**SOD2 < 50%**		**SOD2 > 50%**		
HPV genotype (*)	**Category**	**N**	**(%)**	**N**	**(%)**	**P value**
HPV-16	No	59	(54.6)	49	(45.4)	0.799
	Yes	10	(58.8)	7	(41.2)	
HPV-18	No	64	(54.2)	54	(45.8)	0.458
	Yes	5	(71.4)	2	(28.6)	
HPV non-16/non-18	No	67	(55.8)	53	(44.2)	0.656
	Yes	2	(40.0)	3	(60.0)	
High risk HPV	No	54	(53.5)	47	(46.5)	0.497
	Yes	15	(62.5)	9	(37.5)	

(*) HPV-16 (n = 17), HPV-18 (n = 7); HPV-11 (n = 3); HPV-6 (n = 3); HPV-35 (n = 1); HPV-39 (n = 1). HPV co-infection has been registered in four cases: HPV-6, 11 (n = 2); HPV 6, 11 and 16 (n = 1); HPV-16, 18 and 39 (n = 1).

Table 3 shows the regional lymph node status according to the clinical and pathological variables. In the bivariate analysis, metastatic node was associated with palpable suspicious lymph node in the clinical exam (P < 0.001), tumor size (P = 0.045), histological grade (P = 0.013), perineural invasion (P < 0.001), vascular invasion (P = 0.010), tumor depth (P = 0.002) and SOD2 expression (P = 0.002). All these variables were included in the exploratory logistic regression (Table 4), which identified the following independent predictive factors of lymph node metastasis: palpable suspicious nodes (OR = 8.9; 95% CI: 2.7 – 29.2), tumor depth greater than 5 mm (OR = 11.6; 95% CI: 1.4 – 97.1), perineural invasion (OR = 9.6; 95% CI: 2.7 – 33.6) and SOD2 expression greater than 50% (OR = 3.4; 95% CI: 1.1 – 10.1).

**Table 3:** **Number and percentage of cases with and without regional lymph node metastasis according to clinical and pathological variables**

		**No lymph node metastasis**		**Lymph node metastasis**		
**Variable**	**Category**	**N**	**(%)**	**N**	**(%)**	**P value**
Age	< 50 yo	26	(65.0)	14	(35.0)	0.682
	50 – 59 yo	27	(65.9)	14	(34.1)	
	≥60 yo	21	(56.8)	16	(43.2)	
Palpable suspicious regional	No	48	(84.2)	9	(15.8)	<0.001
Lymph node	Yes	24	(40.7)	35	(59.3)	
pT (TNM)	pT1a	7	(100.0)	0	(0.0)	0.045
	> pT1a	67	(60.4)	44	(39.6)	
Pattern of invasion	Pushing	6	(46.2)	7	(53.8)	0.368
	Infiltrating	60	(61.9)	37	(38.1)	
Histological grade	Grade 1	13	(86.7)	2	(13.3)	0.013
	Grade 2	30	(71.4)	12	(28.6)	
	Grade 3	31	(50.8)	30	(49.2)	
Perineural invasion	No	59	(75.6)	19	(24.4)	<0.001
	Yes	9	(26.5)	25	(73.5)	
Vascular invasion	No	55	(68.8)	25	(31.2)	0.010
	Yes	13	(40.6)	19	(59.4)	
Invasion of *corpora cavernous*	No	14	(66.7)	7	(33.3)	0.303
	Yes	28	(50.9)	27	(49.1)	
Invasion of *corpora spongiosum*	No	3	(75.0)	1	(25.0)	1.000
	Yes	50	(60.2)	33	(39.8)	
Invasion of urethra	No	26	(66.7)	13	(33.3)	0.436
	Yes	14	(56.0)	11	(44.0)	
Tumor depth	≤5 mm	18	(90.0)	2	(10.0)	0.002
	>5 mm	44	(51.2)	42	(48.8)	
HPV-16	No	64	(62.1)	39	(37.9)	1.000
	Yes	10	(66.7)	5	(33.3)	
HPV-18	No	68	(61.3)	43	(38.7)	0.255
	Yes	6	(85.7)	1	(14.3)	
SOD2 expression	<50%	49	(75.4)	16	(24.6)	0.002
	>50%	25	(47.2)	28	(52.8)

**Table 4:** **Predictive factors for regional lymph node metastasis according to the exploratory logistic regression**

**Variable**	**Category**	**N**	Adjusted OR (*)	**95% CI**	**P value**
Palpable suspicious regional lymph node	No	49	1.0	Reference	
	Yes	55	8.9	2.7 – 29.2	<0.001
Tumor depth	≤5 mm	20	1.0	Reference	
	>5 mm	84	11.6	1.4 – 97.1	0.023
Perineural invasion	No	71	1.0	Reference	
	Yes	33	9.6	2.7 – 33.6	<0.001
SOD2 expression	<50%	57	1.0	Reference	
	>50%	47	3.4	1.1 – 10.1	0.029

(*) Number of outcomes included in the analysis: 44 (regional lymph node metastasis).

OR: Odds ratio 95% CI: 95% confidence interval.

Table 5 summarizes the patient’s distribution according to the four predictive factors of inguinal lymph node metastasis found in the multivariate model.

**Table 5:** **Distribution of patients according to the combination of the predictive factors of inguinal lymph node metastasis found in the multivariate model**

**Palpable suspicious lymph node**	**Tumor depth**	**Perineural invasion**	**SOD2 expression (>50%)**	**Patients at risk**	**Patients with inguinal lymph node metastasis**	
				**n**	**n**	**(%)**
No (*1)	≤5 mm	Absent	-	9	0	(0.0)
		Absent	+	4	0	(0.0)
		Present	-	2	0	(0.0)
		Present	+	0	NA	NA
	>5 mm	Absent	-	15	2	(13.3)
		Absent	+	10	2	(20.0)
		Present	-	2	0	(0.0)
		Present	+	7	5	(71.4)
Yes (*2)	≤5 mm	Absent	-	2	0	(0.0)
		Absent	+	0	NA	NA
		Present	-	1	0	(0.0)
		Present	+	2	2	(100.0)
	>5 mm	Absent	-	19	7	(36.8)
		Absent	+	12	8	(66.7)
		Present	-	7	7	(100.0)
		Present	+	12	11	(91.7)
All cases (*3)	≤5 mm	Absent	-	11	0	(0.0)
		Absent	+	4	0	(0.0)
		Present	-	3	0	(0.0)
		Present	+	2	2	(100.0)
	>5 mm	Absent	-	34	9	(26.5)
		Absent	+	24	10	(41.7)
		Present	-	9	7	(77.8)
		Present	+	19	16	(84.2)

NA: Data not available since any case was observed in this situation.

(*1) Eight cases were not included in this group because they did have either tumor depth or perineural information. SOD2 expression was positive in three of them. All those cases had no lymph node metastasis.

(*2) Four cases were not included in this group because they did have either tumor depth or perineural information. SOD2 expression was positive in one of them. All those cases had no lymph node metastasis.

(*3) Two cases with unknown clinical status of inguinal nodes were included in this group. Both cases had tumor depth > 5 mm, no perineural invasion, positive SOD2 expression and no lymph node metastasis.

## Discussion

To the best of our knowledge, this is the first study analyzing the expression of SOD2 in a large series of penile carcinoma samples. SOD2 is one of three distinct superoxide dismutases isoforms found in mammals. This protein is found generally in the mitochondrial matrix and is an evolutionary conserved enzyme in a variety of organisms. Superoxide dismutases act as part of the cellular antioxidant system protecting the redox sensitive cellular machinery from damage induced by reactive oxygen species (ROS). This enzyme’s activity affects important cellular processes including cell growth, proliferation and differentiation. As a consequence the role of SOD2 in cancer is complex and multifactorial. In fact, the exact role of SOD2 and redox state in cancer onset and progression remains poorly understood. Interestingly, accumulating evidence suggest that reducing oxidative stress levels by increasing SOD2 expression may represent a double-edged sword in tumor development. Reducing oxidative stress may have anti-tumoral effect by preventing DNA damage. This is supported by the observation that SOD2 expression can reduce the malignant potential of several transformed cell lines [10,16]. Conversely, it can be anticipated that SOD2 up-regulation may reduce the level of ROS in tumor cells. This may prevent the accumulation of life incompatible levels of DNA and other macromolecules damage, protecting neoplastic cells from intrinsic cell death mechanisms and favoring their survival and spread [8,9].

Analysis of SOD2 expression conducted in solid tumor samples including colorectal [17,18], gastric and esophageal [19,20], oral [21], lung [22-24], brain [25], cervical [11] and skin [26] carcinomas have often associated its up-regulation with metastasis and poor disease outcome. The molecular mechanisms underlying increased SOD2 and metastasis include the alteration of several cellular pathways. It has been observed that SOD2 overexpression increases matrix metalloproteinases (MMPs)-1, −2, −3, −7, −10, −9 and −11 mRNA levels. In the case of MMP-1 this has been attributed to the activation of the Ras/MAP/extracellular signal-regulated kinase signaling cascade. In the same study SOD2 upregulation was associated with enhanced metastatic potential of fibrosarcoma cells in an animal model [27]. Since MMPs play a critical role in the metastatic process it can be argued that the association between increased SOD2 and poor prognosis observed in certain tumors may be attributed to elevated MMP production/activity. A recent study showed that SOD2 expression was sufficient to overcome ROS mediated growth arrest in prostate carcinoma cells [28]. Besides it was observed that elevated SOD2 levels confer resistance to ROS mediated anoikis in mammary epithelial cells in a process dependent on NFκB activation [29]. Since anoikis resistance is essential for tumor metastasis the anti-anoikis activity of SOD2 implicates this enzyme in the metastatic process. We can, therefore, speculate that increased SOD2 levels may confer an adaptive advantage to tumor cells favoring disease establishment and progression.

We have previously reported that SOD2 is differentially expressed between normal and HPV immortalized keratinocytes [30]. Furthermore, we have recently shown that SOD2 protein expression level is a potential biomarker for the characterization of different stages of cervical neoplasia, which is etiologically linked with infection with high-risk HPV types [11]. A proportion of penile cancers are also associated with HPV, mostly HPV16. In fact, recent evidence suggests that these viruses, particularly HPV16, may be associated with up to 48% of all penile tumors [31].

In the present study no association between SOD2 expression and HPV infection was observed. HPV DNA was detected in 20.8% of the samples analyzed which is below the HPV positivity data reported by others [31,32]. This result is not due to quality of the DNA recovered from the paraffinized specimens since >98% of them tested positive for globin. The low proportion of HPV-positive specimens detected probably reflects the fact that in our study most of the samples analyzed are of SCC of the usual histological type, which is less frequently associated with HPV infection. Using exploratory logistic regression we observed that SOD2 expression in more than 50% of the cells was an independent predictive factor of lymph node metastasis as were other well documented clinical-pathologic variables such as palpable suspicious nodes, tumor depth >5 mm and perineural invasion [33-36].

Although SOD2 expression was clearly an independent predictive factor of inguinal lymph node metastasis in the multivariate model, its real contribution in clinical practice remains to be determined. Table 5 demonstrates that tumor depth information is able to determine itself the presence of node metastasis, regardless the status of inguinal node in the clinical exam and perineural invasion status. Our results show that SOD2 expression has some value by increasing the risk of metastasis when the tumor depth was greater than 5 mm. All cases of inguinal metastasis, but two, occurred when the tumor depth was greater than 5 mm. Interestingly, the two cases in which lymph node metastasis was observed when tumor depth was less than 5 mm had positive SOD2 immunoexpression. Coincidently, those cases had palpable suspicious lymph nodes in inguinal clinical exam. The implication of SOD2 expression in thin penile tumors is uncertain and should be interpreted with caution because a limited number of cases in this particular setting were observed. However, the potential clinical implications of this observation as well as the contribution of SOD2 assessment in clinical practice warrants further studies.

## Conclusions

SOD2 expression predicts regional lymph node metastasis in usual type squamous cell carcinomas of the penis. Further studies are needed to determine the clinical implication of this factor.

## Additional files

Additional file 1:
**Immunohistochemical analysis of SOD2 Expression in penile samples.** Representative immunoreactivity of SOD2 in normal penile epithelium (A) and usual penile squamous cell carcinomas (B-D). Less than 50% of stained cells were observed in (B) while (C) and (D) showed more than 50% of stained cells. Magnification: 200x. Additional file 2:
**Frequency of HPV types in penile SCC samples.** Frequency of HPV types in penile SCC samples.

## Abbreviations

HPV: Human papillomavirus
ROS: Reactive oxygen species
SOD2: Superoxide dismutase 2 or manganese superoxide dismutase
