# Supplementary figures and images for: SOD2 immunoexpression predicts lymph node metastasis in penile cancer
Source: BMC Clin Pathol. 2015 Mar 3;15:3. doi: 10.1186/s12907-015-0003-7 (PMC4350326; doi:10.1186/s12907-015-0003-7)

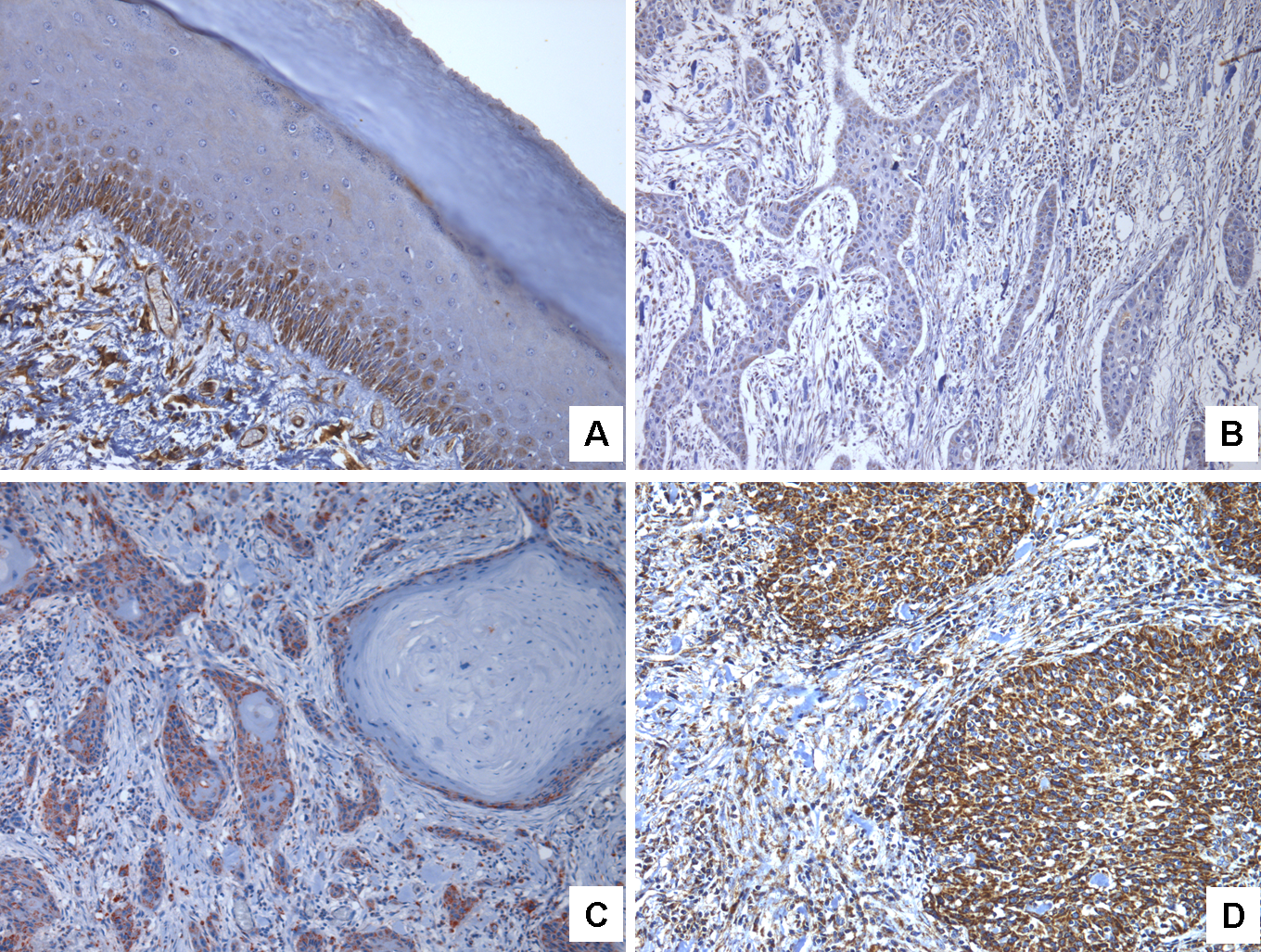

Supplement: Additional file 1: — Immunohistochemical analysis of SOD2 Expression in penile samples. Representative immunoreactivity of SOD2 in normal penile epithelium (A) and usual penile squamous cell carcinomas (B-D). Less than 50% of stained cells were observed in (B) while (C) and (D) showed more than 50% of stained cells. Magnification: 200x. [file 12907_2015_3_MOESM1_ESM.tiff]
