# Supplementary material for: SOD2 immunoexpression predicts lymph node metastasis in penile cancer
Source: BMC Clin Pathol. 2015 Mar 3;15:3. doi: 10.1186/s12907-015-0003-7 (PMC4350326; doi:10.1186/s12907-015-0003-7)
Supplement: Additional file 2: — Frequency of HPV types in penile SCC samples. Frequency of HPV types in penile SCC samples. [file 12907_2015_3_MOESM2_ESM.docx]

| Frequency of HPV types in penile SCC samples | |
| --- | --- |
| **Variable** | **Patients (%)** |
| **HPV genotyping** |  |
| HPV16 | 15 (11.9%) |
| HPV16, 18, 39 | 1 (0.8%) |
| HPV 18 | 6 (4.8%) |
| HPV35 | 1 (0.8%) |
| HPV6, 11 | 2 (1.6%) |
| HPV6, 11, 16 | 1 (0.8%) |
| NEGATIVE | 100 (79.4%) |
|  |  |
